# Melatonin suppresses the antiviral immune response to EMCV infection through intracellular ATP deprivation caused by mitochondrial fragmentation

**DOI:** 10.1016/j.heliyon.2022.e11149

**Published:** 2022-10-19

**Authors:** Mariko Kikuchi, Miki Kadena, Haruka Fukamachi, Takashi Takaki, Shohei Matsui, Sumire Hoashi-Takiguchi, Hirobumi Morisaki, Nataša Trtić, Mina Mori, Mie Kurosawa, Momoe Itsumi, Takahiro Funatsu, Atsuo Sakurai, Seikou Shintani, Hiroki Kato, Takashi Fujita, Yasubumi Maruoka, Hirotaka Kuwata

**Affiliations:** a Department of Oral Microbiology and Immunology, Faculty of Dentistry, Showa University, 1-5-8 Hatanodai, Shinagawa-Ku, Tokyo 142-8555, Japan; b Department of Special Needs Dentistry, Division of Community-Based Comprehensive Dentistry, Faculty of Dentistry, Showa University, 2-1-1 Kitasenzoku, Ohta-ku, Tokyo, 145-8515, Japan; c Department of Special Needs Dentistry, Division of Dentistry for Persons with Disabilities, Faculty of Dentistry, Showa University, 2-1-1 Kitasenzoku, Ohta-ku, Tokyo, 145-8515, Japan; d Division of Electron Microscopy, Showa University, 1-5-8 Hatanodai, Shinagawa-Ku, Tokyo 142-8555, Japan; e Division of Periodontology and Oral Medicine, Division of Periodontology and Oral Medicine, Department of Dental Medicine, Faculty of Medicine, University of Banja Luka, Banja Luka 78000, Republic of Srpska, Bosnia and Herzegovina; f Department of Pediatric Dentistry, Faculty of Dentistry, Showa University, 2-1-1 Kitasenzoku, Ohta-ku, Tokyo, 145-8515, Japan; g Department of Pediatric Dentistry, Tokyo Dental College, Tokyo, 101-0061, Japan; h Institute of Cardiovascular Immunology, University Hospital Bonn, University of Bonn, Bonn, 53105, Germany; i Laboratory of Molecular Genetics, Institute for Frontier Life and Medical Sciences, Kyoto University, Kyoto, 606-8507, Japan

**Keywords:** Innate immunity, Inflammation, Antiviral immunity, Melatonin, Neuro-immunology, Cytokines, Mitochondria

## Abstract

Melatonin, a sleep hormone derived from the pineal gland, has an anti-inflammatory effect on the immune system in addition to modulating the brain nervous system. Previous studies have shown that melatonin suppresses signaling pathways downstream of multiple pattern recognition receptors on the innate immune cells during pathogen infection, but the specific mechanism of suppression has not been well understood. Using an encephalomyocarditis virus (EMCV) infection model in macrophages, we investigated the effects of melatonin on the antiviral response in innate immunity and found that melatonin attenuated the uptake of viral particles into macrophages. Furthermore, melatonin suppressed cytoskeletal regulation by decreasing ATP production by mitochondria. Finally, in an *in vivo* infection experiment, we also found that melatonin administration partially exacerbated the infection in the mouse brain. These results suggest that melatonin may have an inhibitory effect on excessive inflammation by suppressing cytoskeletal regulation in the innate immune system, but also suggest that suppression of inflammation may lead to insufficient protection against EMCV infection *in vivo*.

## 1 Introduction

Melatonin, produced by the pineal gland, acts as a regulator not only for circadian rhythms but also for the immune system [1, 2]. In our previous report, we showed that melatonin functions as an immune regulator of LPS-induction in macrophages through the inhibition of the Toll-like receptor (TLR) 4 signaling pathway, resulting in the suppression of IL-6 and other TRIF-dependent pathways [3]. Importantly, melatonin suppressed multiple signaling pathways and cellular functions such as cytoskeletal dynamics, resulting in decreased clearance by bacterial phagocytosis. Other studies have also shown the inhibitory role of melatonin in inflammatory response related to bacterial infection *in vivo* and *in vitro* [4, 5]. In addition to bacterial infections, the role of melatonin in viral infections is also attracting attention. A previous study showed that melatonin decreased oxidative stress and viral replication, thereby increasing the survival in mice infected *in vivo* with Venezuelan equine encephalitis (VEE), a positive-sense single-stranded RNA virus [6]. Another paper showed that melatonin decreased TLR3-dependent gene expression via inhibition of NF-kB activity in macrophages infected *in vitro* with a respiratory syncytial virus, a negative-sense single-strand RNA virus, while the relationship between melatonin and NF-kB signaling remains unclear [7]. Thus, it has been suggested that melatonin plays a role in regulating the inflammatory response to viral infections. Since the emergence of the pandemic of novel coronavirus disease 2019 (COVID-19), the efficacy of melatonin in reducing the excessive inflammatory symptoms caused by the infection has been investigated. Several research papers have shown its efficacy against excessive inflammation in lethal COVID-19 patients [8, 9, 10].

The single-strand RNA virus encephalomyocarditis (EMCV) which causes myocarditis and encephalitis in various animals including mice has been frequently used as a model for signal transduction research in antiviral immunology, and as a viral RNA genomic stimulus in the study of Toll-like as well as cytosolic receptors such as retinoic acid-inducible gene I (RIG-I)-like receptors family (RLRs) [11, 12]. Recent research of virus recognition by the RLRs signaling pathway has been more influential in the study of innate immunity than on the TLRs signaling pathway [13, 14]. The signaling mechanism by which the melanoma differentiation-associated gene 5 (MDA5), a member of the RLR family, recognizes EMCV-containing viral RNAs is well characterized. In response to virus infection, MDA5 triggers innate immune signaling via the mitochondrial-associated adaptor protein, forming a scaffold that stimulates inflammatory responses such as IFN-β [15, 16].

In the present study, we aimed to elucidate the cellular mechanisms by which melatonin suppresses the innate immune response to viral infection using an EMCV infection model. In the process, we especially examined the relationship between mitochondrial energy production and the suppression of innate immunity. Finally, we investigated the immunological significance of the changes in the mouse body caused by the suppression of the antiviral response specifically for EMCV infection.

## 2 Material and methods

### 2.1 Reagents and cell culture

Melatonin was purchased from Nacalai Tesque (Kyoto, Japan). Murine macrophage cell line, RAW264.7, was cultured in RPMI1640 supplemented with 5% FBS. Transfecting (PH)-PLCδ-GFP plasmid (kindly gifted by Dr. Greg Fairn, Dalhausie Univeristy, Canada) into cells was performed with Neon Transfection System (Thermo Fisher Scientific, Massachusetts, USA). For the Neon transfection, RAW 264.7 cells were transfected with the mixture of plasmid DNA and resuspension buffer and then electroporated with the setting of 1680 V, 20 ms, 1 pulse. Cells were transferred to glass coverslip with RPMI1640 supplemented with 10% FBS for 2 days before microscope observation. For optimal mitochondrial condition, cells were used for all experiments after 3 days of cellular passage. For the hypoxia experiment, RAW264.7 cells were pretreated in an anaerobic chamber for 6 h at 36.5 °C (O_2_ < 0.1%). The cells were then treated with melatonin (1 mM) and infected by EMCV for 16 h. Polyinosinic-polycytidylic acid (poly (I:C), LMW) was purchased from InvivoGen (California, USA). Lipofectamine 3000 (Thermo Fisher Scientific) was used for lipofection for poly (I:C). For immunoblotting, anti-mouse IL-1β antibody (rabbit monoclonal, ab234437, Abcam, Cambridge, UK) and anti-pan actin antibody (mouse monoclonal, ACTN05(C4), ab3280, Abcam) were used.

### 2.2 Viruses

EMCV was prepared by infecting BHK cells at an MOI of 1. Medium containing produced viruses was centrifuged at 3000 rpm for 10 min to pellet the cell debris and stored at -80 °C. The viral titer was assessed by a plaque assay on L929 cells.

### 2.3 Quantitative real-time PCR (qRT-PCR)

For EMCV quantification and cytokine expression analysis, total RNA was prepared with the RNeasy Mini Kit (Qiagen, Hilden Germany) and reverse-transcribed using ReverTraAce qPCR Master Mix (Toyobo, Osaka, Japan) with the mixture of oligo (dT) 20 and random hexamer primers following the manufacturer's protocol. The concentration of cDNA was quantified by a spectrophotometer, and the final concentration was adjusted to 1 μg/μl. For quantification of EMCV RNA, including virus genomes and messenger RNAs, qRT-PCR was performed on a StepOne Plus PCR System using SYBR Green Master Mix (Applied Biosystems, Massachusetts, USA) with specific primers targeting the EMCV capsid coding region. For detecting mtDNA in cytosolic extracts, cytosolic mtDNA was isolated with NE-PER nuclear and cytoplasmic extraction reagents (Thermo fisher scientific) and purified with the QIAquick nucleotide removal kit (Qiagen). Quantification was performed on cytoplasmic fraction for mtDNA and whole-cell extract DNA for GAPDH. Relative cytosolic mtDNA levels were normalized to the total DNA amount. Quantification was performed with the comparative CT method using GAPDH as endogenous control and non-stimulation by Student's t-test with significance set at p < 0.01. The following primer sets were used for GAPDH; 5′-gcacagtcaaggccgagaat-3′ and 5′-gccttctccatggtggtgaa-3’; IFN-β; 5′-ccctatggagatgacggaga-3′ and 5′-ctgtctgctggtggagttca-3’; INOS; 5′-gcttcacttccaatgcaaca-3′ and 5′-ggctggacttttcactctgc-3': EMCV; 5′-ttatagtgccggacctggca-3′ and 5′-cccaagctcccagtgtggtc-3’; mtDNA:5′-gccccagatatagcattccc-3′ and 5′-gttcatcctgttcctgctcc-3'.

### 2.4 Immunofluorescence microscopy

For imaging of mitochondria in live cells, cells were incubated with 100 nM Mito-Tracker Orange CMTMRos (Thermo Fisher Scientific) for 30 min at 37 °C and then washed with HBBS 2 times, then observed with a fluorescent microscope. For imaging of SGs, cells were stained with rabbit monoclonal anti-G3BP antibody (ERP13986, Abcam) after permeabilization with 0.1% TritonX-100. Then cells were washed with PBS and stained with DAPI to visualize nuclei and observed using an epifluorescence inverted microscope (Axiovert200M, Carl Zeiss, Oberkochen, Germany). Analyzing and quantification of fluorescence intensity was done with Zen software (Carl Zeiss). For Airyscan confocal imaging of live mitochondria, high-resolution confocal microscopy was performed on a Zeiss LSM 980 microscope (Carl Zeiss) equipped with a Plan-Apochromat 63X Oil 1.4 NA subsequently analyzed with the Imaris software X64 9.0.0 (Bitplane, Belfast, UK). Mitochondrial detection was done with automatic adjustment and corrected manually.

### 2.5 TEM microscope observation and analysis

Macrophages were cultured on plastic chamber slides (Thermo Fisher Scientific) and pre-fixed in 2.5% glutaraldehyde under moderate conditions at room temperature. The samples were washed four times with PBS for 3 min before incubation with 2% osmium tetroxide for 30 min at 4 °C. After being washed with distilled water for 5 min, the tissues were dehydrated and 100% EtOH for 30 min, finally, the samples were handstand-embedded for 100% resin. The resins were cured at 60 °C for 3 days. The samples were sliced at 70-nm using an ultramicrotome (Ultracut-UCT, Leica, Wetzlar, Germany). After staining of ultrathin sections with uranyl acetate and lead citrate, TEM observation was performed using H-7600 (Hitachi, Tokyo, Japan) [17]. The aspect ratio of each mitochondrion was measured with Image-J software by manually tracing outlines of mitochondria on TEM micrographs and calculated as the result of dividing the value of the major axis by the value of the minor axis. Mitochondria from at least 3 sections of 5 cells from each condition were analyzed.

### 2.6 Measurement of intracellular ATP content

Macrophages were pretreated with 1 mM melatonin for 1 h. Intracellular ATP in macrophages was measured using the Intracellular ATP assay kit v2 (TOYO B-Net Co., Ltd., Tokyo, Japan) based on the luciferin-luciferase reaction according to the manufacturer's instructions. Luminescence was measured with a luminometer (Promega, Wisconsin, USA), and ATP concentration was calculated by using an ATP standard curve.

### 2.7 Mice experiments

Female BALB/c mice (aged 8 weeks; CLEA Japan, Inc., Tokyo, Japan) were divided into 2 groups. One group of mice was given melatonin (10 mg/L) with drinking water that was dissolved in ethanol (final 0.05% v/v in distilled water) before one day of virus infection. A control group was given only ethanol in drinking water. All mice were infected with EMCV (10^5^ pfu per mouse) via intraperitoneal injections. After 3 days of infection, tissues and serum were collected and analyzed for RT-PCR and ELISA. All mice experiments were approved by the Ethics Committee of Showa University (number 16080).

## 3 Results

### 3.1 Melatonin suppresses the antiviral response of innate immunity

To investigate the effect of melatonin on the antiviral immune response, a macrophage cell line (RAW264.7 cell line) was infected with EMCV after treatment with a 1 mM concentration of melatonin for 1h. The response of macrophages to EMCV infection is characterized mainly by the expression of iNOS and type I Interferon [18]. At 16 h post-infection (pi), EMCV infection robustly induced iNOS expression in control macrophages in a dose-dependent manner depending on the multiplicity of infection (MOI), whereas in melatonin-treated, iNOS expression was not observed at high and low MOI (Supplementary Figure 1A). Next, we examined whether melatonin suppressed the expression of type I IFN in macrophages primed with IFN-γ at an MOI of 100 and observed that melatonin significantly suppressed the expression of the IFN-β gene at 16 h pi of EMCV (Supplementary Figure 1B). Thus, we confirmed that melatonin has a role in suppressing the innate immune response during EMCV infection.

### 3.2 Melatonin decreases the accumulation of EMCV RNA in macrophages

**Figure 1:**
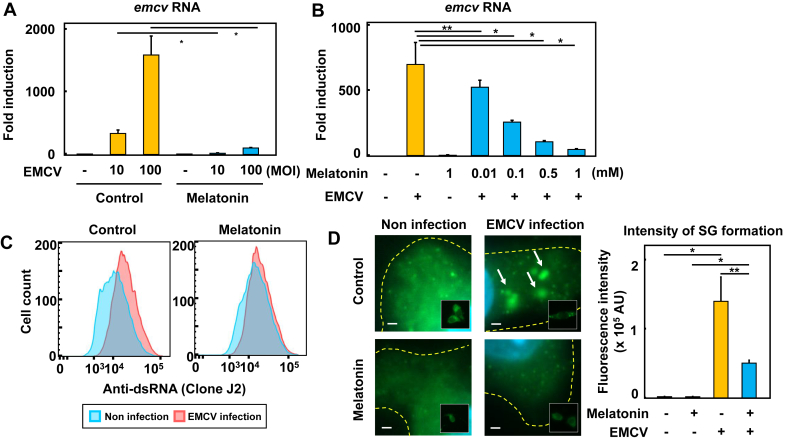
Inhibition of EMCV engulfment and virus particle recognition in macrophages by melatonin (A) Reduction of EMCV engulfment in melatonin-treated macrophages. RAW264.7 cells were infected with EMCV (for 16 h at indicated MOIs) in the presence or absence of melatonin (1 mM for 1 h) (B) Dose-dependent reduction of EMCV uptake into melatonin-treated macrophages. RAW264.7 cells were infected with EMCV (for 16 h at MOI of 100) after pretreatment with various concentrations of melatonin (0.01, 0.1, 0.5, 1 mM for 1 h). Total RNA was extracted and the EMCV RNA including genome RNA and mRNA was determined by RT qPCR with specific primers for EMCV capsid coding region (C) Inhibition of viral dsRNA formation in macrophages by melatonin treatment. RAW264.7 cells were infected with EMCV (at an MOI of 10 at 4 h pi) in the presence or absence of 1 mM melatonin. Cells were stained with anti-dsRNA antibody (J2 clone) and analyzed by flow cytometry. In the control cells, median fluorescent intensities (MFIs) for non-infected and virus-infected cells were 10446 and 15077, respectively. In the melatonin-treated cells, MFIs were 12522 and 14467, respectively (D) Inhibition of stress granules (SGs) assembly in EMCV-infected macrophages by melatonin. RAW264.7 cells were infected with EMCV at MOI of 100 at 6 h pi in the presence or absence of 1 mM melatonin. SGs and nuclei were stained with anti-G3BP antibody (green) and DAPI (blue) respectively (left panel). Images were taken with 100X magnification objective and selected as representative images from 3 independent experiments. Scale bars; 5 μm. The right panel is the quantification of SG formation in cells. The total amount of SG per cell was quantified by selecting the SGs of macrophage manually, measuring the fluorescence intensity, and analyzing the data. Data are expressed as means ± S.D. (n = 3) and all data are representative of three independent experiments. Statistical analysis: ∗p < 0.01, ∗∗p < 0.05 as compared with untreated control.

In our previous studies, we have shown that melatonin has inhibitory effects on the cytoskeleton, such as suppressing actin multimerization in macrophages [3]. Therefore, we hypothesized that melatonin may reduce the uptake of viral particles and analyzed changes in the intracellular amount of viral genomic RNA by quantitative PCR. The results showed that in control macrophages, EMCV RNA was present in large amounts at MOI of 10 and 100 after 16 h pi. However, in melatonin-treated macrophages, the amount of RNA was significantly reduced (Figure 1A). We also found that melatonin significantly suppressed viral RNA in a dose-dependent manner (Figure 1B). Importantly, melatonin significantly suppressed RNA accumulation even at the lowest concentration (0.01 mM).

The initial event in signaling during EMCV infection is the replication of single-stranded genomic RNA in host cells, which triggers antiviral signaling in immune cells [19]. Double-stranded (ds) RNA is synthesized during their replication cycle and is essential for viral immune induction [20]. We hypothesized that melatonin inhibits viral uptake into macrophages after binding to the cell surface, which leads to the induction of antiviral immune response. To test this hypothesis, we measured the change in dsRNA levels in the early stages of infection by flow cytometry. Using anti-dsRNA antibodies, we compared the formation of intracellular dsRNA in macrophages infected with EMCV. In control cells, dsRNA antibody binding was observed after EMCV infection, while the change in fluorescence intensity was less pronounced in melatonin-treated cells (Figure 1C). These data suggest that melatonin inhibited the internalization of EMCV, resulted in reduced viral replication in macrophages. Next, we analyzed the formation of stress granules (SGs) in the cytoplasmic region of macrophages after EMCV infection. EMCV induces the transient formation of SGs, which are aggregates of ribonucleoproteins, in the cytoplasm of infected host cells and serves as an essential platform for the subsequent antiviral response [19]. Ras-Gap SH3 domain binding protein 1 (G3BP1) is a well-characterized SG marker molecule, which is often used to study the subcellular distribution of virus-induced SGs and plays an important role in the expression of type I interferons. Therefore, to compare whether melatonin alters the dynamics of SGs in cytoplasms of macrophages, we examined the accumulation of G3BP1. As expected, melatonin reduced the magnitude of SG formation in EMCV-infected macrophages (Figure 1D). These data indicated that melatonin treatment caused macrophage cells to become unresponsive to EMCV infection.

### 3.3 Melatonin alters mitochondrial morphology in macrophages

**Figure 2:**
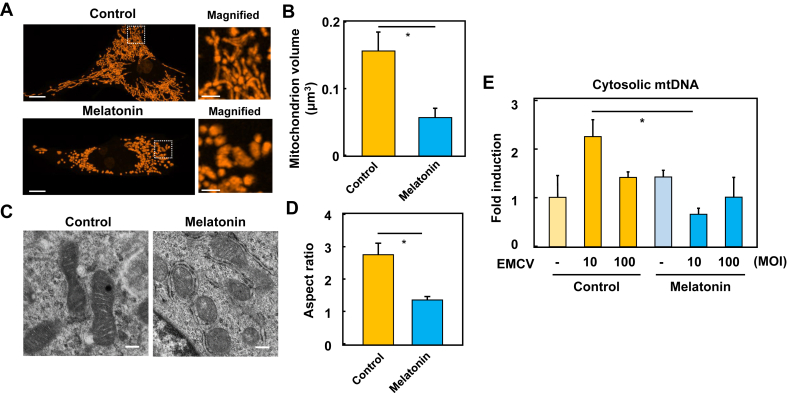
Altered mitochondria morphology and mitochondria DNA in melatonin-treated macrophages (A) High-resolution imaging of live mitochondria in macrophages. RAW264.7 cells were treated with 1 mM melatonin for 1 h, stained with MitoTracker Orange, and observed with confocal laser scanning microscopy with an incubation chamber to control temperature and CO_2_. Scale bars; 5 μm. Higher magnification of mitochondrial morphology is represented in the micrograph (right panel); scale bar; 2 μm average mitochondrial size in macrophages (B) Quantification of melatonin-induced alterations in mitochondrial morphology. The volume of individual mitochondria was measured with software using high-resolution images taken for panel A (C) Electron microscopy of structural alteration in the mitochondria and endoplasmic reticulum of RAW264.7 cells with or without 1 mM melatonin. Scale bars; 200 nm (D) The ratio of major axis per minor axis of each mitochondrion in TEM images of panel C was calculated as the result of dividing the value of the major axis by the value of the minor axis (E) Suppression of cytosolic mtDNA release in melatonin-treated macrophages. RAW264.7 cells were infected with EMCV at MOI of 10 and 100 for 16 h with or without 1 mM melatonin. Cytosolic mtDNA was collected and assessed by qPCR with specific primers for mtDNA. mtDNA levels were normalized to internal control. *gapdh* was used as an internal control for PCR. ∗p < 0.01 as compared with untreated control.

In considering the mechanism of the suppression of anti-viral response by melatonin, we examined the role of mitochondrial respiration. Under the hypoxic condition, the expression of *ifn-β* and *il-6* in response to viral stimuli was significantly suppressed by melatonin compared with that of GAPDH mRNA (Supplementary Figure 2A and B). In addition, the suppression of viral load in macrophages was also inhibited by hypoxia (Supplementary Figure 2C). These data indicate that the inhibitory effect of melatonin on the antiviral response is dependent on mitochondrial function via mitochondrial aerobic respiration. The relationship between mitochondria and antiviral immunity is well-known, with mitochondria functioning as a platform for virus recognition [21]. Mitochondrial membrane proteins that regulate mitochondrial dynamics, such as mitofusins, are important signaling molecules for viral infection [22, 23]. Therefore, to analyze whether melatonin alters mitochondrial morphology, macrophage cells were stained with MitoTracker, and then mitochondrial morphology was observed by high-resolution live-cell imaging techniques. In the control macrophages, the filamentous shape of mitochondria was frequently observed, but when melatonin was distributed, the mitochondria morphology showed clumped features (Figure 2A). To quantify the changes in mitochondrial morphology, three-dimensional (3D) morphometric analysis was used to compare the relative mitochondrial volume of control and melatonin-treated cells. The mitochondria of melatonin-treated cells showed a smaller volume ratio compared to the control mitochondria (Figure 2B), suggesting that melatonin induced the fission of mitochondria. Thus, our results suggest that melatonin alters mitochondrial morphology through the dynamics of fission and fusion.

### 3.4 TEM observation confirms changes in the mitochondrial morphology of macrophages

To further confirm the results of the live-cell 3D imaging, transmission electron microscopy (TEM) analysis was performed to evaluate the mitochondria of both melatonin-treated and control cells. As a result, the morphological feature of mitochondrial clumping similar to 3D live cell imaging was observed in melatonin-treated cells by TEM (Figure 2C) and at the same time, the aspect ratio of mitochondria was reduced in melatonin-treated cells compared to control cells (Figure 2D), suggesting that mitochondria in melatonin-treated cells are blob forms, rather than tubular forms seen in healthy cells. Moreover, as shown in the right panel of Figure 2C, melatonin increased the frequency of contact between the rough endoplasmic reticulum (RER) and mitochondria. Previous reports have shown that the endoplasmic reticulum (ER) membrane ubiquitin ligase Gp78 intensifies RER-mitochondria contacts through the degradation of mitofusin 2 [24] and modulation of RER-mitochondria interaction is essential for intracellular calcium homeostasis [25]. Therefore, these physical associations between ER and mitochondria in melatonin processing may support the idea that Ca^2+^ influx is involved in ATP synthesis.

In addition, a previous study has examined how certain viral infections release mitochondrial DNA (mtDNA) into the cytoplasm, elevating the type I interferon response and conferring antiviral immunity [26]. Therefore, we next stimulated macrophages with EMCV and analyzed the release of cytosolic mtDNA by qPCR with a specific primer to mtDNA. In control macrophages, the cytosolic release of mtDNA increased at an MOI of 10. In contrast, cells treated with melatonin did not release cytosolic mtDNA at any MOI condition (Figure 2E). These data indicate that melatonin inhibits virus uptake into cells at an early stage, resulting in the attenuated release of cytoplasmic mtDNA by EMCV infection.

Additionally, since the release of mtDNA into the cytosol typically trigger the NLRP3 inflammasome activation [22], we tested whether melatonin inhibits the IL-1β production that subsequently follows inflammasome activation using immunoblotting and ELISA assays. The results showed that melatonin treatment suppressed by approximately 50% both the production of pro-IL1β and p17 fragment, which is secreted form of IL-1β, during EMCV infection from LPS-primed RAW264.7 cells (Supplementary Figure 3A and B). The release of IL-1β into the culture supernatant was similarly suppressed by approximately 50% (Supplementary Figure 3C) in the melatonin-treatment macrophages.

### 3.5 Melatonin decreases intracellular ATP

**Figure 3:**
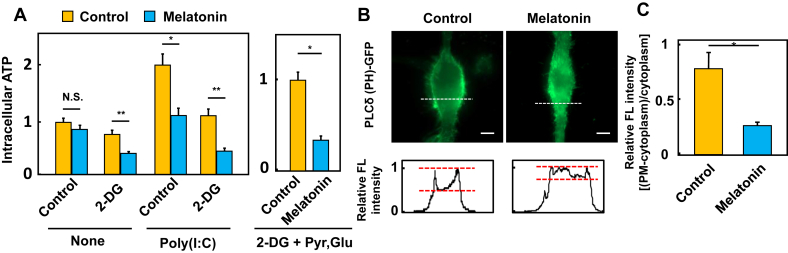
Melatonin-induced dysregulation of mitochondrial function in macrophages (A) Effect of metabolic regulation on intracellular ATP production in macrophages with or without melatonin. RAW264.7 cells were incubated with 1 mM melatonin for 1 h following treatment with 10 mM 2-DG (left panel) with or without 1 mM sodium pyruvate and 2 mM glutamate (right panel). Cell stimulation was performed by transfection with 50 μg/ml poly (I:C) and analyzed after 16 h. The data are shown as the means ± S. D. of three samples and statistical analysis of data was performed using the student's T-test. Similar data were obtained from three independent assays (B) Plasmalemmal localization of PIP2 in melatonin-treated macrophages. RAW264.7 cells transiently expressing PLCδ (PH)-GFP, a biosensor for PIP2. After incubation for 2 days, live cells were treated with or without 1 mM melatonin for 1 h immediately before using an inverted epifluorescent microscope through a 100X objective lens. Scale bars; 5 μm (C) Quantification of plasmic membrane fluorescence intensity in control- or melatonin-treated macrophages expressing the GFP biosensor. Values were obtained from images of panel B by dividing the difference between plasmalemmal and cytoplasmic fluorescence by the cytoplasmic fluorescence, thus accounting for differential levels of expression and photobleaching. Data were expressed as means ± S. D. Experiments were conducted at least three times. Statistical analysis: ∗p < 0.01, ∗∗p < 0.05 compared to untreated control, N.S. not significant (A, C).

Mitochondria are regarded as the energy factories of the cell, as ATP is produced by oxidative phosphorylation within mitochondria and intracellular glycolytic pathways. A previous report has shown that a rapid reduction of intracellular ATP in macrophages by nigericin treatment mediates mitochondrial depolarization and induces IL-1β expression and inflammasome activation [27]. In this study, we examined whether melatonin affects the maintenance of ATP levels in macrophages. The glucose analog, 2-deoxy-D-glucose (2-DG, 10 mM), impairs glycolysis and pyruvate production, drives intracellular ATP production by oxidative phosphorylation (OXPHOS) in mitochondria and decreases intracellular ATP in aerobic condition. In melatonin-treated cells, intracellular ATP was more reduced in the presence of 2-DG (Figure 3A left panel). Interestingly, stimulation with poly (I:C) increased intracellular ATP. This is probably because activation of NF-kB by poly (I:C) induces M1 differentiation of macrophages that depend on glycolysis for ATP production [28].

Supplementation with pyruvate (1 mM) and glutamine (2 mM) is often used to upregulate ATP production by OXPHOS and to compensate for the decrease in intracellular ATP in normal cells. Since supplementation with pyruvate and glutamine more potently inhibited intracellular ATP production, melatonin may have a significant influence on the process of ATP production in the activated state OXPHOS under an anaerobic environment (Figure 3A right panel).

### 3.6 Melatonin disturbs PtdIns(4,5)P_2_ distribution in the plasma membrane

Intracellular ATP is crucial for cellular functions such as receptor-mediated endocytosis and cytoskeleton dynamics [29]. Therefore, we hypothesized that melatonin-induced depletion of intracellular ATP is the mechanism that suppresses the immune response to EMCV and viral replication. Our previous report showed that melatonin treatment destabilizes actin filament polymerization and inhibits phagocytosis [3]. Considering that melatonin rapidly decreases intracellular ATP that is associated with cellular functions including membrane traffic, it is necessary to examine whether melatonin correlates with membrane traffic in macrophages. It is also well known that phosphoinositide plays an important role in the coordination of cytoskeletal organization and membrane trafficking [30]. Therefore, to examine whether melatonin alters phosphoinositide metabolism, the subcellular distribution of PtdIns(4,5)P_2_ (PIP2) was visualized with the PLCδ(PH)-GFP [31]. In control cells, PLCδ(PH)-GFP was found along the entire plasma membrane, where PIP2 is most abundant, however, after melatonin treatment, plasmalemmal PIP2 distribution was not detected (Figure 3B), suggesting that the intracellular PIP2 concentration was reduced (see Figure 3C for quantification of normalized fluorescence).

### 3.7 Effect of melatonin on EMCV infectivity in the brain and interferon production in serum

**Figure 4:**
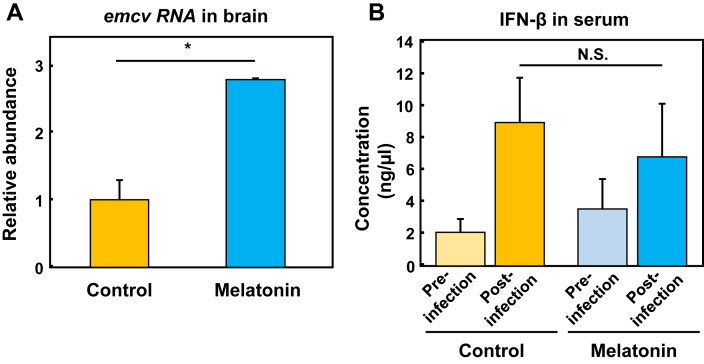
Melatonin administration to mice enhances the EMCV infection in the brain (A) After the administration of melatonin (10 mg/L) by drinking water, Balb/c mice (8-weeks-old females) were intraperitoneally injected with EMCV (10^5^ pfu per mice). Then, tissues were collected 3 days after virus infection. RNA was collected from the tissues and synthesized cDNA was subjected to real-time PCR with specific primers for *emcv* (B) Sera were collected at the time of tissue obtaining, and the IFN-β concentration in serum was determined by ELISA. Data are mean ± S. E. of three mice in a single experiment as a representative of three independent experiments (A, B). ∗p < 0.05 compared to untreated controls. N.S. not significant.

Previously, melatonin has been shown to reduce oxidative stress and increase the NO production in the brains of mice, suggesting a potential antiviral agent using the Venezuelan equine encephalitis virus, VEE [6]. In this study, we examined the effect of melatonin on EMCV infectivity in the brains of BALB/c mice by oral administration in drinking water. Compared to control mice, a significant increase in the amount of virus in the brains of melatonin-treated mice was observed (Figure 4A), but changes in survival rate and symptoms such as paralysis were not evident. In addition, there was a trend toward an increase in the amount of virus in the heart tissue of mice treated with melatonin, but no statistically significant change was observed (data not shown). The effect of melatonin in EMCV infection was shown to be opposite to the trend in VEE infection [6, 32]. At the same time, the production of IFN-β in the serum of melatonin-treated mice was also examined, but no significant difference was observed (Figure 4B). Thus, different type of virus may vary in their effect on melatonin in the brain.

## 4 Discussion

We have previously reported that melatonin attenuates inflammatory signaling pathways in macrophages using an analysis method called Immuno-navigator, which utilizes a transcriptional factor binding site prediction system [3]. The results showed that melatonin preferentially downregulates interferon regulatory factors (IRFs) and signal transducers and activators of transcription (STATs) which are crucial to antivirus response. During that analysis, Melatonin was also predicted to modulate mitochondrial function by inhibiting the binding activity of hypoxia-inducible factors (HIFs), which mediate adaptation to hypoxia. Several studies have already indicated that melatonin works as an antioxidant and protects mitochondria homeostasis against oxidative stress [33]. Probably, the regulatory action of melatonin is complicated during pathogen infection. In this study, we have shown that melatonin suppresses gene expression of type I IFN and iNOS in macrophages during EMCV infection.

The effect of melatonin on the mitochondrial morphology of macrophage cells was transient, and upon the removal of melatonin from the culture supernatant, the mitochondria reverted to their original tubular morphology after 1 h (Supplementary Figure 4A). Induction of mitophagy by carbonyl cyanide m-chlorophenylhydrazone (CCCP, 20 μM) which irreversibly disrupts the mitochondrial membrane potential, resulted in the release of cytochrome c from the mitochondria, whereas no cytochrome c release was observed with melatonin, indicating that the action of melatonin is protective for the mitochondria (Supplementary Figure 4B). The metabolism of melatonin is rapid and mitochondrial morphology may be precisely regulated by various enzymes and degradation mechanisms in the cell.

In addition, there was no inhibitory effect of melatonin on poly (I:C) transfection (Supplementary Figure 5A). Transfection of poly (I:C) into macrophages caused SG formation and activated RIG-I and MDA5 signaling, but at least melatonin treatment did not affect SG formation itself. In contrast, poly (I:C) treatment caused activation by both TLR3 and RIGI/MDA5 in macrophages, so the effect of melatonin on the RIG-I/MDA5 pathway alone remains unclear. Melatonin treatment attenuated inflammasome induction by EMCV infection in macrophage (Supplementary Figure 3). Our findings are consistent with previous studies that have shown that melatonin mitigates the activity of the NLRP3 inflammasome via ROS-related intracellular signaling pathways [34].

The effect of melatonin may differ depending on the type of virus. Both EMCV and VEE are known to cause viral encephalitis. However, in contrast to previous results with VEE [32], our experiments with *in vivo* infection with EMCV showed that melatonin increased the amount of EMCV virus in the brain. Different viruses have different tropisms, which possibly reflect different effects of melatonin on IFN-β-producing cells *in vivo*, such as fibroblasts and endothelial cells. Alternatively, the innate immune system in the brain has recently been known to have different properties than those outside the brain [35]. In that case, the effects of melatonin may have a different impact on macrophage-like cells such as microglia in the brain [36].

This study suggests that the melatonin-induced attenuation of the antiviral immune response to EMCV in the innate immune system is due to the inhibition of viral uptake into cells and that the mechanism is intracellular ATP depletion through the inhibition of reversible mitochondrial activity by melatonin. Regarding viral infection and mitochondrial morphology, it has been reported that Hepatitis C virus infection induces mitochondrial fission and mitophagy, resulting in the attenuation of antiviral response [37]. In contrast, Influenza A virus (IAV) infection causes mitochondrial elongation and reduces IAV replication [38]. A variety of molecules involved in mitochondrial fusion and fission has been studied, and it is expected that the mechanism of mitochondrial regulation by melatonin will be elucidated in more detail by studying the functional changes of these molecules in the future. Such studies may provide new therapeutic strategies for various intractable inflammatory diseases, such as autoimmune inflammatory diseases and sepsis, as well as severe viral infections.

## Declarations

### Author contribution statement

Mariko Kikuchi and Miki Kadena: Performed the experiments; Analyzed and interpreted the data; Wrote the paper.

Haruka Fukamachi, Takashi Takaki, Shohei Matsui, Sumire Hoashi-Takiguchi, and Hirobumi Morisaki: Performed the experiments; Analyzed and interpreted the data.

Nataša Trtić, Mina Mori, Mie Kurosawa, Momoe Itsumi, and Takahiro Funatsu: Analyzed and interpreted the data.

Atsuo Sakurai, Seikou Shintani, Hiroki Kato, Takashi Fujita, and Yasubumi Maruoka: Conceived and designed the experiments; Contributed reagents, materials, analysis tools or data.

Hirotaka Kuwata: Conceived and designed the experiments; Analyzed and interpreted the data; Wrote the paper.

### Funding statement

Professor Hirotaka Kuwata was supported by 10.13039/501100001691Japan Society for the Promotion of Science [18K09558].

Miki Kadena was supported by 10.13039/501100000646Japan Society for the Promotion of Science [18K17264].

Mariko Kikuchi was supported by 10.13039/501100001691Japan Society for the Promotion of Science [21K21097].

### Data availability statement

Data will be made available on request.

### Declaration of interest's statement

The authors declare no conflict of interest.

### Additional information

No additional information is available for this paper.

## Appendix A Supplementary data

SupFig_1007.pptx SupFig_1007.pptx
