# Supplementary material for: Melatonin suppresses the antiviral immune response to EMCV infection through intracellular ATP deprivation caused by mitochondrial fragmentation
Source: Heliyon. 2022 Oct 19;8(10):e11149. doi: 10.1016/j.heliyon.2022.e11149 (PMC9593192; doi:10.1016/j.heliyon.2022.e11149)
Supplement: SupFig_1007.pptx [file mmc1.pptx]

## Slide 1
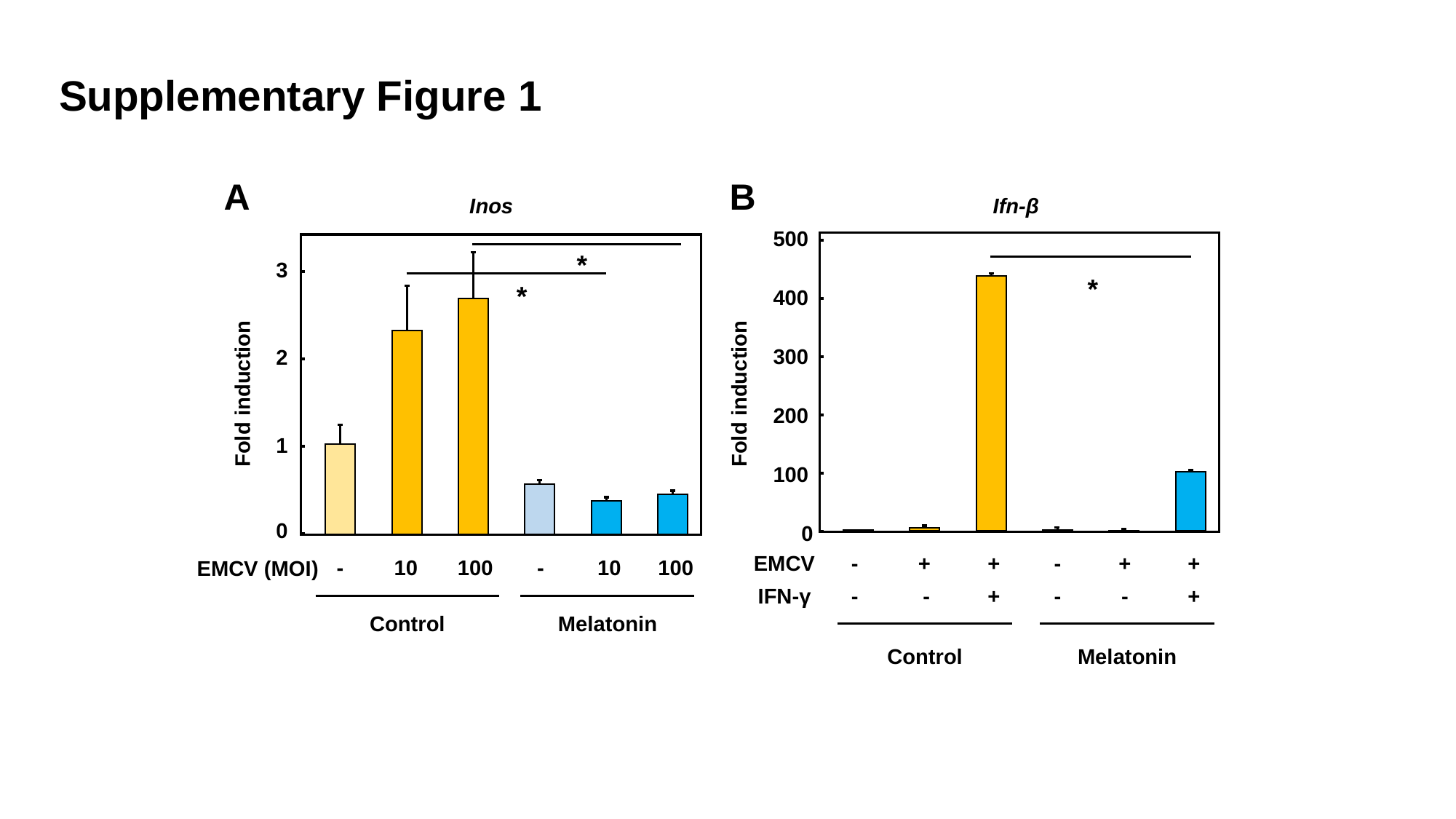

Supplementary Figure 1
A
B
Inos
*
3
2
1
0
*
Fold induction
-
10
100
-
10
100
EMCV (MOI)
Control
Melatonin
Ifn-β
500
400
300
200
100
0
*
Fold induction
EMCV
-
+
+
-
+
+
IFN-γ
-
-
+
-
-
+
Control
Melatonin

## Slide 2
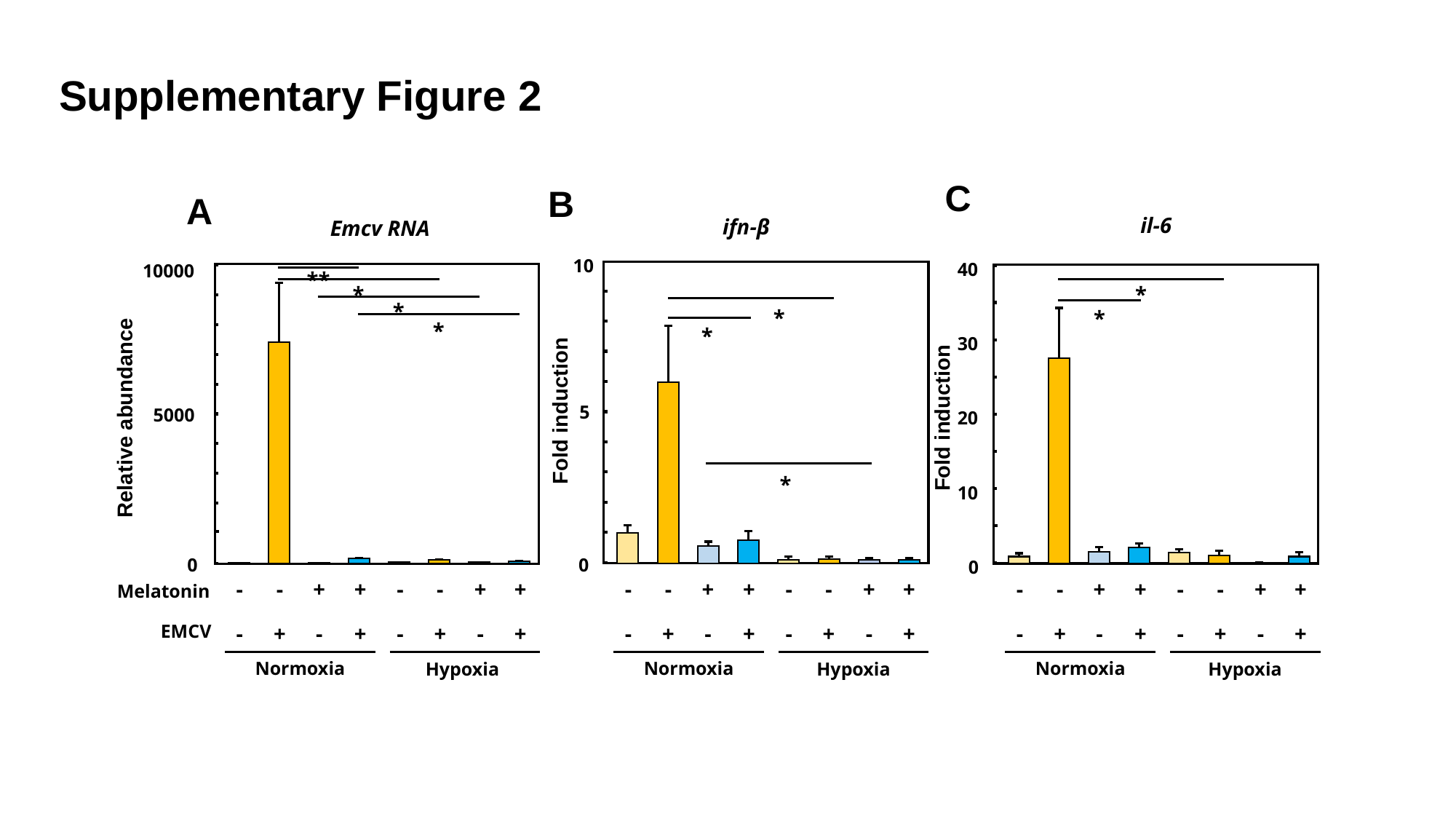

Supplementary Figure 2
C
B
A
il-6
40
30
20
10
0
*
*
-
-
+
+
-
-
+
+
-
+
-
+
-
+
-
+
Normoxia
Hypoxia
ifn-β
Emcv RNA
10000
5000
0
**
*
*
*
-
-
+
+
-
-
+
+
Melatonin
EMCV
-
+
-
+
-
+
-
+
Normoxia
Hypoxia
10
5
0
*
*
*
Fold induction
Relative abundance
Fold induction
-
-
+
+
-
-
+
+
-
+
-
+
-
+
-
+
Normoxia
Hypoxia

## Slide 3
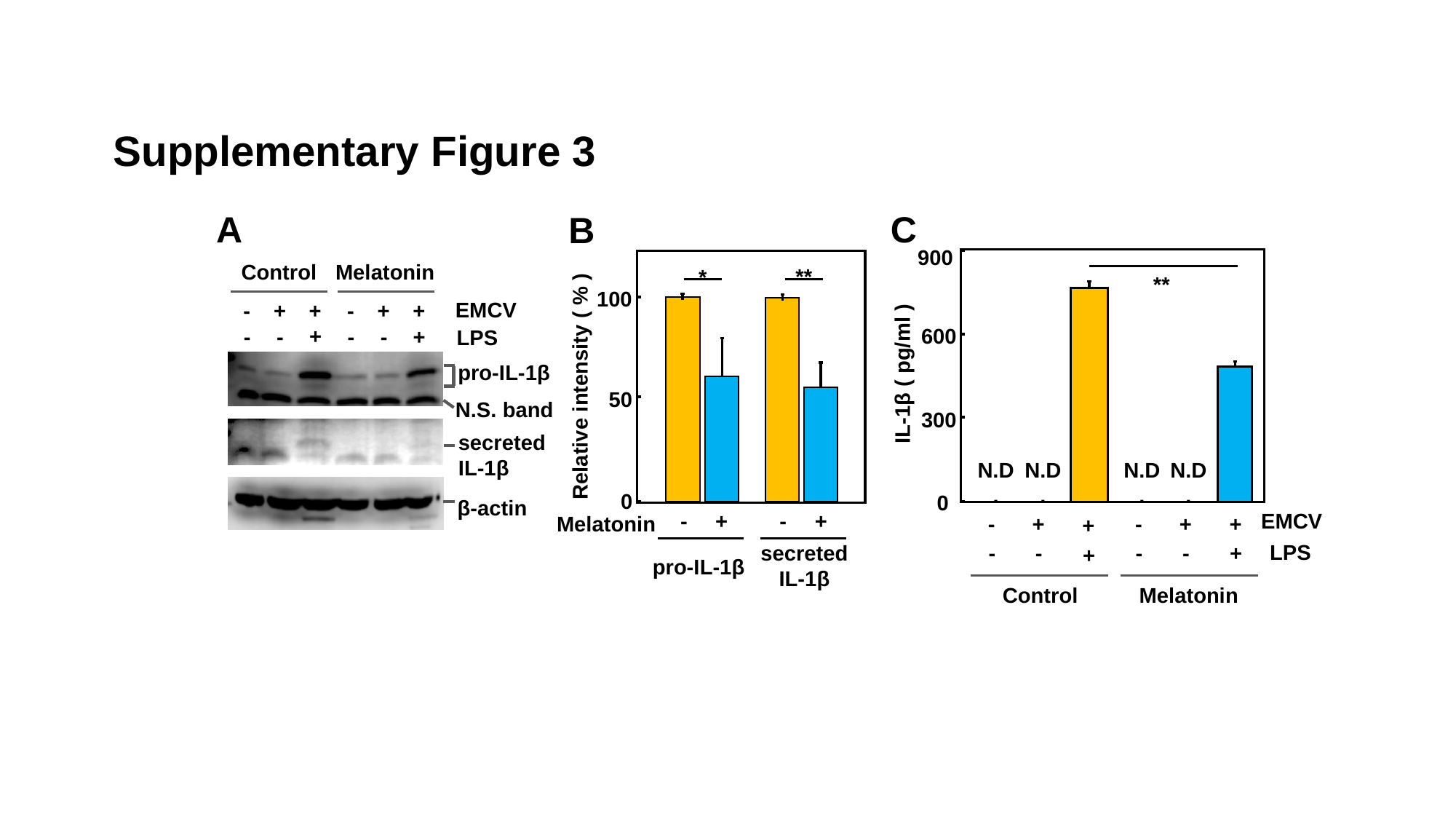

Supplementary Figure 3
A
C
B
900
600
300
0
**
N.D.
N.D.
N.D.
N.D.
-
+
+
-
+
+
-
-
+
-
-
+
LPS
Melatonin
Control
**
*
100
50
0
Control
Melatonin
EMCV
-
+
+
-
+
+
-
-
+
-
-
+
LPS
pro-IL-1β
IL-1β ( pg/ml )
Relative intensity ( % )
N.S. band
secreted IL-1β
β-actin
EMCV
-
+
-
+
Melatonin
secreted
IL-1β
pro-IL-1β

## Slide 4
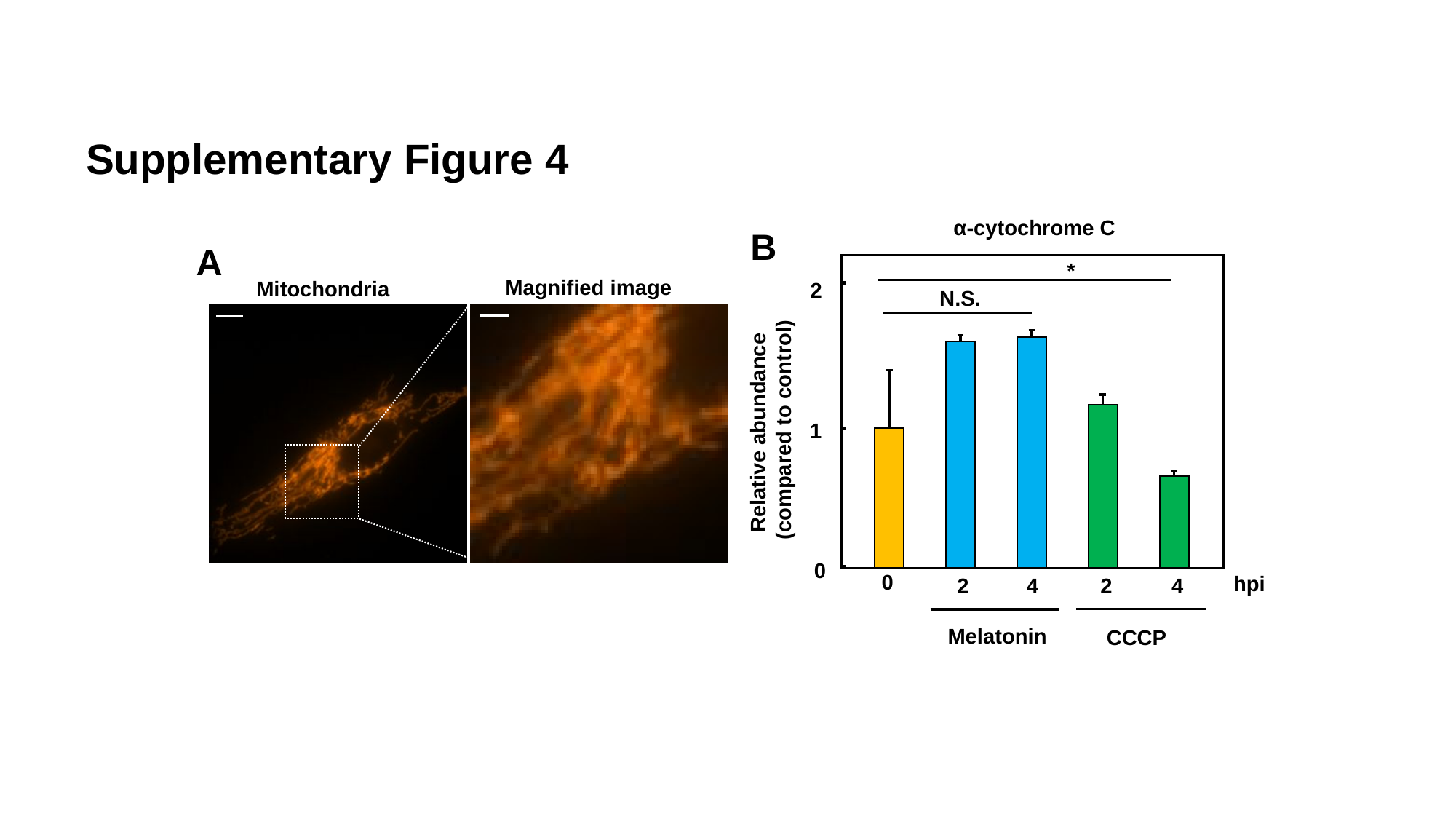

Supplementary Figure 4
α-cytochrome C
B
A
*
1
0
Magnified image
Mitochondria
2
N.S.
Relative abundance (compared to control)
0
hpi
2
4
2
4
Melatonin
CCCP

## Slide 5
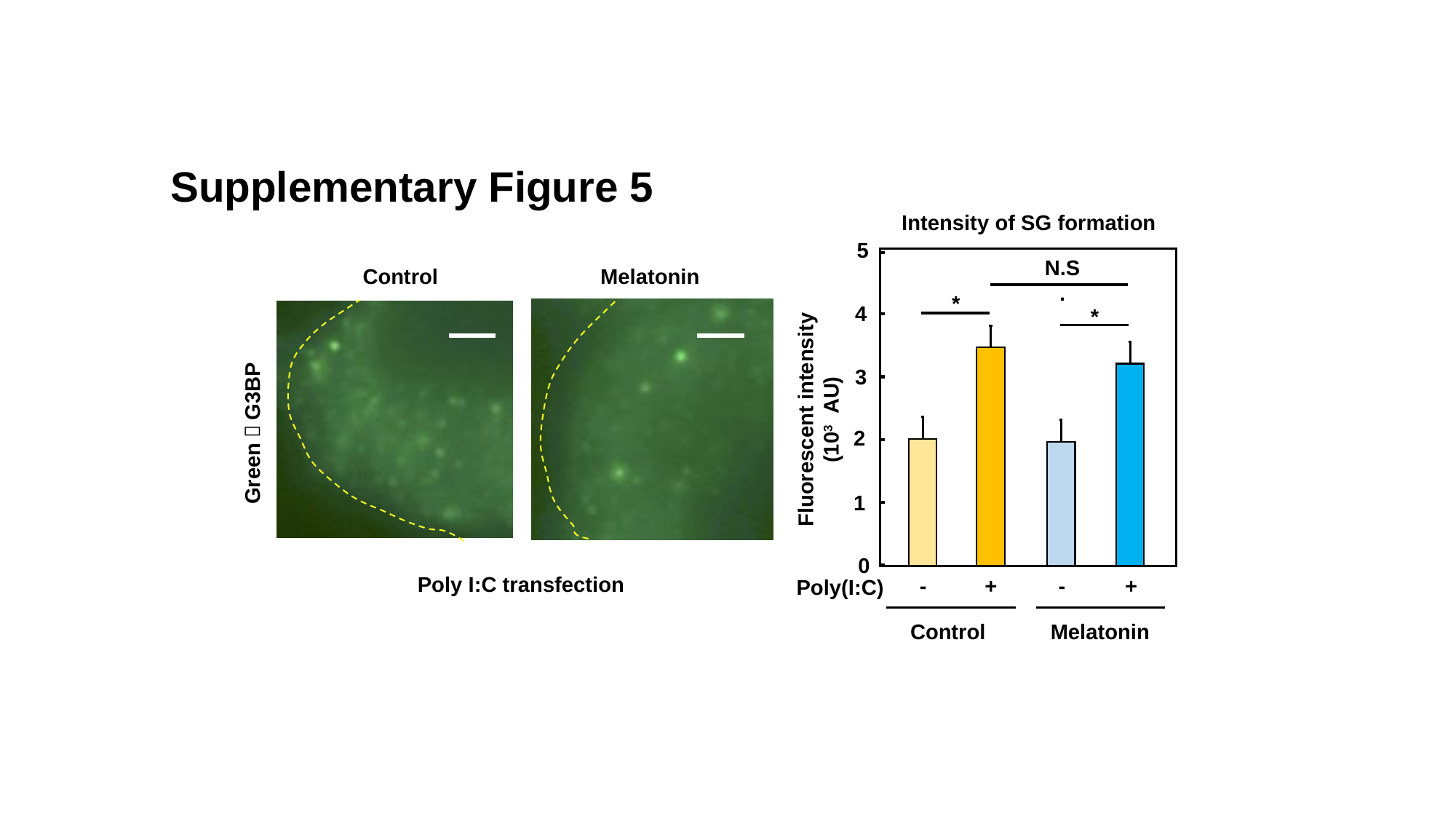

Supplementary Figure 5
Intensity of SG formation
5
N.S.
Control
Melatonin
*
4
3
2
1
0
*
Fluorescent intensity
(103 AU)
Green：G3BP
Poly I:C transfection
-
+
-
+
Poly(I:C)
Control
Melatonin
